# Health care professionals’ experiences and perceptions of health promotion through the health dialogue intervention in the scania region, Sweden: a qualitative interview study

**DOI:** 10.1186/s12875-023-02133-2

**Published:** 2023-09-04

**Authors:** Sara Alenius, Kjell Olsson, Ena Thomasson, Lina Magnusson

**Affiliations:** 1 Department of Health Sciences, Faculty of Medicine, Lund University, Box 157, Lund, 221 00 Sweden; 2 Nutritional Epidemiology, Department of Clinical Sciences Malmö, Lund University, Malmö, SE-21428 Sweden; 3 Department of Healthcare Management, Region Skåne, Kristianstad, 291 89 Sweden

**Keywords:** Content analysis, Health dialogue, Health promotion, Primary care, Sweden

## Abstract

**Background:**

Cardiovascular disease and type 2 diabetes are among the largest public health challenges in Sweden. Research indicates that a healthy lifestyle can prevent most cases. The health dialogue is an evidence-based public health programme for primary care with positive results in several regions of Sweden. This study aimed to describe health care professionals’ experiences and perceptions of health promotion through the health dialogue intervention during the pilot phase in the Scania region of Sweden.

**Methods:**

The study consists of 12 individual interviews with health care professionals educated in the health dialogue method, implementing the intervention in Scania. Qualitative content analysis with an inductive approach was used.

**Results:**

The analysis resulted in 10 sub-categories and the four main categories: A more health-promoting mindset would benefit primary care; Empower individuals; Facilitate sustainable lifestyle changes; Challenges, tools and support for the implementation of the health dialogue. One overarching theme emerged: “*Health dialogue, a potential start of a paradigm shift in Swedish primary care”*.

**Conclusions:**

Conclusions imply that the health dialogue is a well-structured method with tools to make health promotion and primary prevention an integrated part of primary care. A respectful and motivating approach during the health dialogue is recommended. It is important to have an ongoing discussion about the approach among the health care professionals. Incorporating the Health Belief Model in the health care professionals’ education in the method could increase the focus on self-efficacy during counselling, which could favour the participants’ change process.

**Supplementary Information:**

The online version contains supplementary material available at 10.1186/s12875-023-02133-2.

## Background

In Sweden, non-communicable diseases cause about 90% of all deaths [1, 2] and cardiovascular disease (CVD) is the predominant cause of mortality, responsible for about 35% of all deaths in Sweden [1, 2]. CVD is also the largest contributor to the burden of disease, measured in disability-adjusted life-years [3–5]. Diabetes is responsible for nearly 4% of all deaths in Sweden and is among the most common causes of death and disability [4]. Type 2 diabetes highly increases the risk of CVD [6]. CVD and type 2 diabetes are preventable through improved lifestyle factors [3, 7–10]. Scania is the southernmost region of Sweden, consisting of 33 municipalities and about 1.4 million inhabitants [11]. A recent public health survey showed that unhealthy lifestyle factors were common in Scania [12]. One of the regional goals by 2030 is to improve public health and quality of life for all, through increased health equality and interventions to promote a healthier lifestyle [13].

### The health dialogue method

The health dialogue is a public health program aimed to reduce CVD incidence and prevalence and has been implemented in several regions across Sweden since the 1980s. Long-term follow-up studies have shown a significant reduction in premature mortality [14–16]. A cost-effectiveness analysis from the region of Västerbotten conducted from 1990 to 2006 found that the cost per quality-adjusted life-year (QALY) from a societal perspective was 62 Euro, compared to the Swedish threshold value of 48 000 Euro per QALY, which makes the method very cost-effective [17]. Through the health dialogue, selected age groups from the population are invited to individual and standardized health examinations and dialogues to prevent CVD. The method includes blood sampling, measuring blood lipids and fasting blood glucose, and measuring blood pressure, body mass index and waist-hip ratio. The participants fill out an extensive online questionnaire about their lifestyle, encompassing dietary habits, physical activity, tobacco use, alcohol consumption and social factors such as relationships, work situation, sleep and stress. Additionally, there is one section about the family history of CVD and diabetes. The results of the questionnaire, biomarkers, and body measurements are displayed in an individual graphic health curve illustrating 11 risk factors for CVD, automatically created by a software. The participants then come to the primary care clinic to see a health dialogue practitioner for an approximately one-hour motivational interview based on their health curve [18].

The method applies both a low- and a high-risk strategy as all inhabitants are invited independent of health status. Participants identified as being at high risk are offered follow-up visits according to their specific needs [18]. The health dialogue practitioner is also encouraged to reach out to local stakeholders, for example, the municipality, grocery stores, pharmacies, and sports associations, for collaborations to facilitate health promotion on a community level [18]. The Health Belief Model is a long-established public health theory that explains individual health behaviour change [19–22]. The health dialogue method was developed in the 1980s and inspired by the Health Belief Model (H, Lingfors, personal communication 22 April 2021). According to the theory, individuals will take action to improve their health if they perceive themselves as susceptible to a condition, disease, or other health problem that they believe can have a serious impact on them. It is important that the individual understands which actions are considered feasible, and where they think the benefits exceed the barriers. In addition, they need to believe that the action can reduce their susceptibility. To act, self-efficacy is also needed [22]. Self-efficacy describes one’s own perceived ability to carry out the act or change a certain behaviour [19–22].

To our knowledge, one qualitative study investigating Swedish public health nurses with many years experience in the method of health dialogue in a primary care setting in a northern region was found [23]. Another study about a similar method [24] and three additional articles about the method of health dialogue in other settings [25–27]. This study can contribute to the knowledge about health care professionals’ experiences and perceptions of learning the health dialogue method and start working with it in a primary care setting.

## Method

This study aimed to describe primary care professionals’ experiences and perceptions of health promotion through the health dialogue method during the pilot phase of the intervention in the Scania region.

### Study setting and sampling strategy

A four-month pilot phase of the health dialogue was conducted in the Scania region of Sweden between October 2020 and January 2021, in 11 out of the total 158 primary care clinics in the region. A pilot phase coordinated by the Department of Healthcare Management in the Scania region was chosen to test the method and implementation strategy before a political decision was made about long-term implementation [18]. Primary care in Sweden covers the entire population, is tax-financed and is the first instance of health care. All inhabitants choose which clinic to be listed at no matter the geographical area, or if the clinic is private or public. All primary health care clinics in Scania were invited to participate in the pilot phase. 50 of 158 clinics showed interest and the 11 clinics were chosen for the pilot phase to reach maximum variation regarding rural/urban area, public/private clinics, size, geographical area and care need index [28]. Registered nurses, physiotherapists and dietitians, at the 11 primary care clinics conducted the health dialogues as part of their work. The health care professionals received a five-day formal education in the health dialogue method, including motivational interviewing. The health care professionals received a method guide consisting of a summary of the latest evidence on areas covered in the health questionnaire and the health curve, used for the health dialogue and guidance for follow-up on detected health risks [18]. They also had the possibility to contact the Department of Healthcare Management for support. A new software was built for the pilot phase but was delayed for about two months. Thus, the health care professionals were offered to start with a printed version of the questionnaire where they manually calculated the results from the health questionnaire to produce the graphic health curve. All inhabitants born in 1980 and listed at the participating clinics in the pilot phase were invited to a health dialogue. Each primary care clinic had 1–3 health care professionals working part-time with the health dialogue.

In this study, purposive sampling was applied to obtain experiences and perceptions of the central phenomena by including different professions and both public and private clinics [29–31]. The inclusion criteria were registered primary care professionals (nurses, physiotherapists, and dietitians) who had been working as health care professionals within the pilot phase. In total, 21 health care professionals at 11 different primary care clinics in eight municipalities, including eight public and three private clinics, met the inclusion criteria and were identified through the Department of Healthcare Management in the Scania region. Invitation letters were sent out to the 21 eligible informants and among these, a total of 12 informants responded and agreed to participate: eight informants from six public clinics and four informants from two private clinics. The clinics included were situated in six municipalities allocated in all geographical areas of Scania (west, Malmö, south and northeast). The different professions participating in the pilot phase were all represented: nurses [7], specialist nurses [2], physiotherapists [2], and dietitians [1]. Nine informants were women and three were men. The average age of the informants was 41 years and ranged from 24 to 71. The average experience of the professionals was 15 years and ranged from one to 50 years. Six informants had previous experience with motivational interviewing before the pilot phase.

### Data collection

The Swedish Ethical Review Authority provided ethical approval for our study, as a part of a larger research project examining the health dialogue method (dnr. 2020–07138). Our study was conducted in accordance with the Helsinki Declaration principles of autonomy, beneficence, non-maleficence, and justice [32]. Informants received an information letter about the study in Swedish, and both written- and oral consent were obtained. A semi-structured interview guide was chosen because it gave a clear structure yet left space for emerging questions during interviews (attached in supplement) [33]. Initially, pilot interviews with two health care professionals were conducted which resulted in minor changes to the interview guide. The interviews were conducted through the encrypted video conferencing platform Zoom. A video conference was chosen because it is as similar to an in-person meeting as possible, compared to telephone interviews, for example [34, 35]. The data was collected during spring 2021 in Swedish and audio-recorded with a mobile phone. The first author (SA) conducted all interviews. Substantive, methodological, and analytical field notes were written during and after each interview. The average length of the interviews was 59 min and ranged from 43 to 83 min. The audio recordings were used for verbatim transcription of each interview. Member checking was done with one informant due to insufficient audio recording quality of the last 20 min of the interview. No substantial new information emerged during the last interviews, which implies that saturation was reached [30].

### Analytical approach

Qualitative content analysis according to Graneheim and Lundman (2004) was applied [36] to understand and describe primary care professionals’ subjective, lived experiences and perceptions of health promotion through the health dialogue. The analytical process started by dividing all of the verbatim transcribed individual interview text related to the aim into meaning units. The meaning units were paragraphs of information referring to a specific topic relevant to the study’s aim and research questions [36] (see Table 1). Then the meaning units were condensed meaning that the text of each meaning unit was shortened but the core of what was said was preserved. After that, each condensed meaning unit was labelled with a code, which allowed the data to be explored in new ways and reflected the key content of each meaning unit [36] (see Table 1). Thereafter the authors SA and LM looked for patterns, similarities, differences, and emerging meanings among all codes from all interviews. The codes were sorted into content areas, then into sub-categories, which were thereafter grouped into categories. In the final step, the categories were interpreted into one overarching theme. Up until the process of coding, everything was conducted in Swedish, which was the mother tongue of the researchers and the informants. The coding and the following steps of the analysis were conducted in English. The quotes used for the presentation were translated into English with some minor grammatical corrections. Finally, sub-categories, categories and the theme were reviewed by authors KO and ET. An audit trail was created during the research process, consisting of methodological and analytical field notes including reflections and ideas during the analytical phase [30, 31].

**Table 1:** Examples of the data analysis process

Meaning unit	Condensed meaning unit	Code	Sub-category	Category
*“*It will have a great effect I think, on those who want help. Because I mean, in the end, it is voluntary and unfortunately, there are probably many who would have needed it who declined. Uhm, but sure I think it can be, it will take time, it probably will, but I think we will see great results in 10–15 years, by starting something like this. I think the burden on health care can decrease, absolutely, I believe so. That people take more responsibility for their health and, kind of, do not need to seek health care because of health problems they get out of a bad lifestyle. So, I absolutely think it will have a positive effect.*”* Informant 10	This can have a great effect. Voluntary so many who need it might decline. Takes time to see results but probably great effect in 10–15 years. Decrease the burden on health care. Make people take responsibility for their health.	Decrease the burden on health care through awareness and lifestyle choices.	Encourage a healthy lifestyle in the whole population	A more health promoting mindset would benefit primary health care
*“*Primarily the participants realize that their current lifestyle does not look so good. Many live in denial, that I am healthy, then there is no reason I will not be healthy in the future. But when they get it on paper, that this is an unhealthy behaviour, regarding heart health, then they have realized that they can change some habits. Many are perceived as motivated to change and also happy to get to know that there is potential for healthier habits.*”* Informant 1	The participants realize that their current lifestyle is not so good. Many live in denial that they feel good now, and do not see any risk of not feeling good in the future. Unhealthy habits on paper give insights. Motivated to change.	Many live in denial but health status on paper gives insights and motivation to change.	A chance to stop and reflect if changes are needed	Empower the participants

## Results

Four main categories emerged, derived from ten sub-categories. The informants’ perceptions and experiences of the pilot phase were marked by the overarching theme *“Health dialogue, a potential start of a paradigm shift in Swedish primary care.”* The results are presented in Table 2. The interviewees, the health care professionals, are referred to as *informants* and the persons they met and conducted the health dialogues with are referred to as *participants*.

**Table 2:** Overview of emerging sub-categories, categories, and the theme in the data analysis

Sub-category	Category	Theme
The health dialogue is health promotion	**A more health-** **promoting mindset would benefit primary health care**	*Health dialogue, a potential start of a paradigm shift in Swedish primary health care*
Encourage a healthy lifestyle in the whole population		
The health care professionals’ new knowledge will benefit patients		
Supportive colleagues are important when primary prevention introduces an additional patient group		
A chance to stop and reflect if changes are needed	**Empower individuals**	
Let the participant guide the dialogue		
Being invited to a health dialogue regularly will give a sense of comfort and a reminder to prioritize health	**Facilitate sustainable lifestyle changes**	
It is important to support the participants in changing their lifestyle habits		
The tools and materials are unique and effective	**Challenges, tools and support for the implementation of the health dialogue**	
Method and competence support are necessary		

### A more health-promoting mindset would benefit primary care

The informants experienced the health dialogue as a clear method to work with health promotion in primary care. By inviting all inhabitants in specific age groups, primary care can potentially reach and introduce a new group of patients, which might lead to early detection of disease. This could benefit both the patient and the health care system. Although, this increases the burden on primary care initially and the intervention needs to be well established on all levels of decision-making and appropriate resources allocated. The intervention has the potential to spread knowledge about lifestyle habits by participants to their families and friends. Local collaborations were identified as a potential development to increase health promotion. The health care professionals described that they incorporated their new knowledge of lifestyle habits into all patient meetings they had to empower patients to make active choices about their lifestyles. This could benefit primary care by potentially delaying and reducing the lifestyle-related burden of disease through improved lifestyle habits.

#### The health dialogue is health promotion

Informants expressed that the health dialogue was a structured way to naturally implement health promotion and primary prevention into primary care and that an increased focus on health promotion and raised awareness about one’s lifestyle can be a way to reduce the burden on the health care system in the long term, both through primary and secondary prevention.

*“In the future, I hope that the health dialogue will be a natural part of health care, for 40-year-olds and 50- and 60-year-olds. This, I believe, will make a major difference as we find many diseases at early stages and persons at risk of developing disease.”* Informant 1.

#### Encourage a healthy lifestyle in the whole population

Informants expressed that the goal of the health dialogue is to raise awareness about health and lifestyle, specifically among the invited participants, but as a secondary consequence, they may spread the awareness to their family and friends as well. Another important part of the intervention is local collaboration to reach the community level. Most of the informants had not started local collaborations during the pilot phase due to a lack of time and local restrictions caused by the Covid-19 pandemic. However, all informants stressed the importance of this part of the intervention and had ideas for future collaborations.

*“We have collaborated with a pharmacy, they distributed information pamphlets to customers about our intervention for two weeks, and they also advertised their health-promoting products. The local grocery store has advertised our intervention, and we started a discussion about their placement of healthy products, but we have not come so far with that yet […] We have contacted the municipality and have had meetings with them […] The public health plan is not updated and needs to be revised, so we said that we would like to be a part of the revision.”* Informant 11.

#### The health care professionals’ new knowledge will benefit patients

Informants experienced that the health-promoting aspect of the health dialogue was ever-present and made them integrate lifestyle questions into their everyday work, thereby benefitting many of their patients. Another example was that the motivational interviewing method helped nurses obtain a lot of information quickly during telephone triage, as it allowed more focus on the patients. An important benefit of the method was that the informants learned to view the patient in a more holistic way than before.

*“I think that you get a completely new understanding of the whole person, by not just focusing on, for example, the medical part, and staring blindly at that because it is all about the holistic view. We have talked a lot about this, that you learn to see the whole person, not just a small area with a problem.”* Informant 10.

#### Supportive colleagues are important when primary prevention introduces an additional patient group

Informants reported that they had support for their work with the health dialogue at their clinics. Supportive management allocated the time needed, allowed the intervention to be as prioritized as the other tasks and had regular follow-ups. At some of the clinics, participants’ colleagues perceived it as inappropriate to carry out the pilot phase during the Covid-19 pandemic. While most informants experienced support from their colleagues, they had an understanding for the fact that introducing health promotion and primary prevention into primary care can bring more work initially as patients will be treated at an earlier stage of their disease, but that it can decrease the burden on the health care system in the long term.

“*The doctors at our clinic are very curious and they ask how the work is proceeding and so on, and I think everyone at the clinic is aware that this can increase the burden on primary care initially but on the other hand maybe we can reduce the burden in the future”* Informant 12.

### Empower individuals

The informants reported that they realized how the health dialogue method could be used to enhance the participants’ sense of empowerment over their health. In motivational interviewing, the health care professional should ask for permission to give information, for example about smoking. This allows the participants to decide which areas they want the dialogue to focus on and they could also choose to avoid certain areas. There was a great variation in the dialogues, in some cases, several risk factors occurred and some focused on addressing potential risk factors while others focused on maintaining and promoting healthy behaviours.

#### A chance to stop and reflect if changes are needed

Informants experienced that all participants benefitted from the health dialogue, irrespective of health status. Perfectly healthy participants got a confirmation that they could continue with their routines, while others with several health risks got a wake-up call, increased awareness of their risks and often realizations about what they needed to change. Some participants misleadingly perceived themselves as healthy and some lived in denial about their health risks, but getting an evidence-based, visual health curve in their hands often gave the participants insight and motivation to change. The health dialogue empowers the participants. After acquiring knowledge about risk factors and getting an increased awareness of their health status the participants could make informed choices about their lifestyle.

*“Being aware, one can make active choices in everyday life. Choose active transportation, walk, bike and so on. When one goes grocery shopping one can make active choices there, there are many alternatives to what one usually buys.”* Informant 9.

Informants explained that many health risks were detected early, which raised awareness among participants about how their everyday lifestyle choices affect their long-term risk of disease. Hence, they got the chance to change their habits gradually, which can be beneficial to achieve sustainable change. Some informants indicated that answering the questionnaire beforehand often gave more truthful answers and mentally prepared the participants for the appointment. In fact, some informants reported that participants who were aware of certain health risks even changed their habits before the dialogue. The informants had different ways of approaching the participants’ health risks, some were guiding and encouraging, while others had a more fear-inducing approach. Although, they all aimed toward informing and empowering the participants.

#### Let the participant guide the dialogue

Informants expressed that the graphic health curve was a pedagogic way to talk about lifestyle habits based on the participants’ results. The informants perceived motivational interviewing as a helpful method for letting the participant guide the dialogue. It was important to adjust the dialogue according to the participant, for example, leaving out the medical terminology to facilitate a conversation without prestige. It was also important, in some dialogues, to point out that the aim was to inform and offer help and let it be completely up to the participant to make their own choices so that they felt empowered instead of forced. Some informants let the participant choose what areas to talk about and some tried to cover all areas.

*“First, I explained to the participant what the health curve is and how it looks, before starting. Then I would show their health curve to them and ask if there is anything specific that they want to focus on or if it is okay if we start from the top to the bottom. Partly because if you had talked about all areas then I think that they felt like the red areas were a little less dramatic because there might also be green areas. If you only focus on the red areas, then it could be perceived as criticism.”* Informant 6.

### Facilitate sustainable lifestyle changes

The informants experienced that the participants had a positive attitude towards the health dialogue, and many expressed a wish to be offered regular health dialogues and to engage with their health care provider outside of being sick. Many informants thought this could help participants in adopting sustainable lifestyle changes. The informants experienced that the amount of support requested to make healthy changes varied greatly among participants. While follow-up visits were one way to provide support, it was not an explicit part of the intervention, unless the health care professionals identified risk factors.

#### Being invited to a health dialogue regularly will give a sense of comfort and a reminder to prioritize health

Informants reported that they had participants who asked about coming back for a new health dialogue in the future. They perceived the participants as interested in the intervention and grateful for the insights it gave them about their health. The informants reflected on the 40-year-old age group and that they are in the midst of life, with the double task of child-rearing and advancing their careers, which can be stressful. The informants also raised that 40 years old are often young enough to identify risks early and prevent non-communicable diseases. However, 50-year-olds may have more time to prioritize their health as their children are older. Some informants indicated that it would ease the transition into retirement and decrease the risk of falling ill soon after retirement if 65-year-olds were included, with a focus on the lifestyle changes retirement often brings. Informants reported that they thought it would benefit most individuals to have specific age groups as well as health dialogues through referral in Scania.

*“I think the participants would appreciate being invited every 10 years. Then they would get a reminder not to forget themselves and to prioritize their health.”* Informant 9.

#### It is important to support the participants in changing their lifestyle habits

Informants stressed the importance of supporting the participants’ behaviour change process. Follow-up visits and referrals to other health care professionals when needed were mentioned as ways to support the participants. Health risks were sometimes associated with social factors, which can be difficult to change. Thus, support was sometimes essential. If the participants were given just one appointment, some of them might change their habits for a few weeks and then fall back into their old habits again. Some informants solved this by offering the participants a follow-up visit after six months, when necessary, to evaluate their lifestyle changes.

*“I had some participants who needed to change their diet and increase their physical activity and they were motivated to change. I offered to contact them again, either by phone or to book a new appointment where I would weigh them and measure their hip-waist ratio again. Almost all of them were positive, just to get some feedback, from someone who actually followed up with you. It is not just empty words that I should try to change my diet so that I can lose that 30 kg overweight that I have that is actually dangerous to me, and then to know that yes, she will call me in six months again and then we will follow-up and see if the changes I have made have been beneficial or not.”* Informant 6.

Some informants indicated that another reason for follow-up was that they found it difficult to cover health goals during the health dialogue, either due to lack of time or because they judged it overwhelming for the participant to talk about goals during the first appointment. Some informants booked a follow-up appointment where they talked about goals or gave the participants a health plan folder where they could fill in their goals themselves.

### Challenges, tools and support for the implementation of the health dialogue

Informants expressed that the health dialogue method had a clear structure, with helpful tools, including biomarkers, the health questionnaire and the software that generated the health curve. This was then followed by a motivational interview based on each participant’s individual health curve. Although the informants were satisfied with the method, there were struggles during the pilot phase, due to both technical issues and limited time. One important facilitating aspect was the method- and competence support offered by the Department of Healthcare Management in the Scania region throughout the pilot phase.

#### The tools and materials are unique and effective

Informants described that the method guide was helpful when they were new to the method. Reading relevant parts of the guide was a good way to prepare for appointments. It was also important to go through the health questionnaire before the appointment to know what factors caused each health risk. Informants experienced that it was challenging to find the reasons behind the health risks in the software compared to the printed version, where they would see the corresponding answers behind an outcome in the health curve, for example, high consumption of red meat which could indicate suboptimal diet in the health curve. Making these connections clearer in the software would make the dialogues easier to conduct.

The health curve was fundamental to the method. A few informants reported that they had previously conducted health talks, similar to the health dialogue but more basic, with patients at their clinics before the pilot phase. They described that the health dialogue method gave a much better structure which made it easier. They pointed out that the health curve and the software were unique and essential, and that the health questionnaire includes more areas than what they had previously experienced.

*“The health curve is very clear, it is really great… It would have been harder to conduct a dialogue like that, just out of thin air and just talk about the areas, here you really get it on print.”* Informant 10.

#### Method and competence support are necessary

The Department of Healthcare Management in Scania has functioned as a method- and competence support, both through the five days formal education of health care professionals and through support during the pilot phase. Informants found it challenging to be part of a pilot because it was hard to estimate how much time would be needed and the start of the software was continuously postponed due to technical issues. Many informants started with a printed version of the health questionnaire during the software delay. Among those who started with the printed version, many experienced that it was time-consuming and difficult to do the calculations and syntaxes behind the outcomes in the health curve manually. Those who waited for the software found it stressful to see the waiting lists and some of them had to work overtime to make up for the time lost. Informants expressed that the method and competence support was accessible and helpful, and that support was crucial, especially at the beginning of the implementation. They also stated that it was positive that someone had an overview of the project and could keep all clinics synchronized.

*“They have been available, and they answered our questions basically the same day […] and wanting to support, it has been a good collaboration.”* Informant 4.

*“Truly great support, really. Nothing feels embarrassing or difficult to ask, truly great collaboration.”* Informant 3.

### Emerging theme: Health dialogue, a potential start of a paradigm shift in swedish primary care

What all emerging categories had in common was the hope that the health dialogue intervention would be the start of a greater change in primary care and a paradigm shift towards an increased focus on health promotion. A more health-promoting mindset, through the health dialogue intervention, would benefit primary care in the sense that it is a cost-effective way to promote health, as well as prevent- and delay lifestyle-related diseases, such as CVD and type 2 diabetes. It has the potential, to reduce the burden on primary care long term. Informants experienced that the health dialogue method empowered the participants to make active choices about their own health, which would benefit both the individual and the health care system. For the health dialogue to help facilitate sustainable lifestyle changes, the health care professionals need to understand the participant’s needs and offer a follow-up visit when necessary. Informants expressed that the health dialogue gave them systematic tools which made the implementation easier, compared to other methods for lifestyle counselling.

## Discussion

The findings from our study indicate that the informants believe in the health dialogue method and the intervention gave them hope for a paradigm shift in Swedish primary care, towards a greater focus on health promotion. Both how to empower the participant, facilitate lifestyle changes, and how the health dialogue method works were important aspects for the informants for the implementation. The findings indicate the importance of letting the participant guide the dialogue and stress the importance of motivational interviewing as a facilitator for a good dialogue. However, informants had different previous experiences with motivational interviewing, and some found it hard to learn the method. A Danish qualitative study on preventive health dialogues in general practice indicated that it can be hard for clinicians to combine motivational interviewing and a health curve tool [24]. A systematic review of motivational interviewing as a tool to support lifestyle change among individuals with increased risk of CVD found that the effectiveness remains uncertain and that more research is needed [37]. Although, another systematic review and meta-analysis on motivational interviewing for lifestyle counselling found promising results [38]. Due to the complexity of counselling lifestyle changes as well as combining a health curve tool with motivational interviewing, it might be advisable to offer the health care professionals regular follow-up training to develop their motivational interviewing skills.

Informants were highly motivated to empower the participants. A systematic review among nurses implies that empowerment is complex [39]. The findings from our study indicate that different approaches were used to address lifestyle factors and health risks during the health dialogue, despite receiving the same education and instructions. Some had a guiding and encouraging approach while others used negative motivation. Similar results were found in a qualitative study on primary care nurses in northern Sweden performing health dialogues [23], where some used a method characterized by a listening, guiding, motivating, and confidence-building approach while others used a method characterized by directing the conversation, pressuring, instilling fear, and demanding responsibility. Both these findings indicate that various approaches are applied by individual health care professionals. However, in accordance with motivational interviewing and the health belief model, the respectful, guiding, motivating and encouraging approach is recommended rather than an intimidating approach. It is advisable to have an ongoing discussion about the most appropriate approach among the health care professionals. In Scania, registered health care professionals (i.e. nurses, physiotherapists, dietitians, occupational therapists, and physicians) are invited to perform health dialogues. During the pilot phase, only nurses, physiotherapists, and dietitians participated. If different professions have different strategies for conducting health dialogues and their respective effects need to be explored in future studies.

Only a few informants had started local collaborations during the pilot phase, although everyone considered it important. A long-term follow-up study of the health dialogue method in Sweden raised community-oriented efforts as one of the success factors [15]. A qualitative study on health behaviour changes in primary care in London indicated the importance of a supportive community context and societal reminders for sustainable lifestyle changes [40]. Additionally, a discussion paper suggests that interventions that require low-level individual agency are more effective and improve health equality compared to interventions that require high-level individual agency [41]. There is not yet a systematic structure on a regional level for local collaborations to facilitate health promotion, therefore a large responsibility falls on the health care professionals and the primary care clinics.

During the dialogues, the informants encouraged the participants’ own suggestions of possible lifestyle changes as well as suggested changes that could decrease their health risks. This was often followed by the participant’s reflections on perceived benefits and barriers of a change, where the informant would explain known, realistic, outcome expectations if the participant was unaware of those. Findings from our study indicate that the dialogues covered the first two steps of the Health Belief Model: (1) Perceived susceptibility to a problem and perceived seriousness of consequences of a problem, which leads to a perceived threat: (2) Perceived benefits of a specified action and perceived barriers to acting, which leads to outcome expectations: (3) Perceived ability to carry out recommended action, which is defined as self-efficacy [19, 22, 42]. The findings revealed that most informants did not talk about self-efficacy during the dialogues. Supporting self-management through a focus on self-efficacy and behaviour change is supported by previous research [43, 44]. A qualitative study from Sweden further supports the importance of self-efficacy for lifestyle changes, through patients’ perspectives [45]. Incorporating the Health Belief Model in the informants’ five days formal education could possibly further improve the education and strengthen communication around self-efficacy during the health dialogues to benefit the participants’ health behaviour change processes.

### Methodological considerations

The sampling variation was achieved by including informants with different professions, sex and ages from eight out of 11 possible, both private and public primary care clinics from six municipalities. A total of 12 out of 21 possible informants provided several perspectives on the central phenomenon. Despite a small sample, no substantial new information emerged during the last interviews which indicated that saturation was reached [31]. The data was collected during the Covid-19 pandemic. Due to local recommendations, all interviews were conducted via Zoom video conferencing to allow real-time face-to-face meetings, which is more likely to provide rich data and rapport building between the interviewer and the participants compared to telephone interviews [34, 35]. However, real-life interviews would probably have made rapport-building easier which might have provided richer data.

Authors SA, KO and ET were knowledgeable about the health dialogue method and LM was experienced in qualitative research methodology. SA and LM conducted the analysis together to strengthen trustworthiness [28, 29]. An iterative data analysis was used and the data was continuously re-examined with increasing insights [29].

This study contributes to the knowledge about different health care professionals’ experiences and perceptions of the health dialogue method and implementation. The findings highlight important aspects experienced by the health care professionals new to the health dialogue method. This can contribute to the planning and implementation of the health dialogue method in other regions and countries.

## Conclusions

Our study indicates that health care professionals implementing the health dialogues are optimistic about the potential of the method to bring an increased focus on health promotion in primary care. The health dialogue is a well-structured method with tools to make health promotion and primary prevention an integrated part of primary care. A respectful and motivating approach during the health dialogue is recommended, and it is important to have a continuous discussion about this among health care professionals. Incorporating the Health Belief Model in the health care professionals’ five days formal education could increase the focus on self-efficacy during counselling, which could favour the participants’ behaviour change process.

### Electronic supplementary material

Below is the link to the electronic supplementary material.

Supplementary Material 1

## Data Availability

The datasets generated and analysed during the current study are not publicly available due to individual privacy but are available from the corresponding author on reasonable request.

## Declarations

### Authors’ information

Researchers’ professional background and preunderstanding is known to influence the research process [46]. Author SA is a PhD student, with a master in public health and registered physiotherapist. SA has a preunderstanding of the primary care context in Sweden from working as a physiotherapist. SA had the five days formal education in the health dialogue method but did not work with health dialogues and did not have a relation to the health care professionals included in the study. Author KO is a PhD student, with a master in public health and registered dietitian. Author ET has a PhD in nutrition and a background as a registered dietitian. KO and ET work at the Department of Healthcare Management in the Scania region, which was the coordinating unit of the pilot phase. Author LM is an experienced researcher within public health and qualitative research methods.

## List of abbreviations

*CVD*: Cardiovascular disease
*QALY*: Quality-adjusted life-year
