# Supplementary material for: Health care professionals’ experiences and perceptions of health promotion through the health dialogue intervention in the scania region, Sweden: a qualitative interview study
Source: BMC Prim Care. 2023 Sep 4;24:173. doi: 10.1186/s12875-023-02133-2 (PMC10476376; doi:10.1186/s12875-023-02133-2)
Supplement: Supplementary file 1 — Supplementary Material 1 [file 12875_2023_2133_MOESM1_ESM.docx]

Interview guide

Interviewer and date:

**Demographic questions:**

Gender; Profession; Age; Primary care clinic; Part of Scania; Professional experience; Previous experience of motivational interviewing; Previous experience of health promotion;

**Health promotion**

What does *health promotion* mean to you?

Can you please describe your experiences of working with the health dialogue?

What do you think will be the effect on public health in Scania by increased emphasize on health promotion in primary care?

**The dialogues**

Can you please describe your experience of inviting participants by mail and call.

- Examples of good experiences
- Examples of difficulties

Can you please describe your experience of the health dialogues?

- Please describe one health dialogue that felt great, in detail.
- Please describe one health dialogue that was difficult, in detail, what was difficult, how did you handle it?

How did you experience the training in motivational interviewing?

- If it was insufficient, how can it be improved?

Did you have any dialogues using an interpreter? Please describe.

Can you please describe how you used the health curve in the health dialogues?

What insights do the participants get during the health dialogues?

- Did many need to get awareness about health risks? How did they react?

What long-term health effects do you think the health dialogue will have on the participants health?

**Organizational aspects**

Can you please describe how the organizational aspects of the intervention worked at your clinic? E.g. inviting, booking, time allocation, collaborations etc.

Can you please tell me about how you think the documentation system worked?

Can you please describe how you experienced your clinic’s participation in the lager research project?

- How was the collaboration with colleagues?
- Was the training about the research project sufficient?

Can you please describe how you experienced the software for the health questionnaire and the health curve?

How did you experience using the printed health questionnaires?

Can you please describe how you experienced the communication materials? Such as the invitation, the invitation in other languages, the health plan, brochures about lifestyle habits, brochures about local recreation activities, posters etc.

Can you please describe how you experienced the health dialogue method guide?

How did you experience the 5 days formal education in the health dialogue method?

Can you please describe how you experienced working with the implementation of health dialogues alongside your regular working tasks?

- How much time and priority where you allowed to allocate to the project? Was it enough?
- How much time and priority where you allowed to allocate to health promotion before the project?

How have collaborations around the project worked at your clinic?

- Have you experienced support from your manager and colleagues?

Can you please describe how you experienced the Department of Healthcare Management as a knowledge- and method support?

- Examples of good experiences
- Ideas for improvement

**Local collaboration**

Can you please describe local initiatives around the clinic to facilitate collaboration and the community perspective of the health dialogue intervention?

- Examples of good experiences
- Examples of difficulties

How much time could you allocate to local collaborations during the project?

What are your ideas for local collaborations in the future?

**Closing questions**

Do you want to continue working with the health dialogue during the long-term implementation?

What do you think is important in long-term implementation?

How do you think health promotion in primary care should be developed in the future?

Health care professionals working with health dialogue can have different professional backgrounds. What effect do you think it will have on primary care that more health care professionals get trained in talking to patients about all lifestyle factors?

Is there anything else you would like to add?
